# The Agreement Between Virtual Patient and Unannounced Standardized Patient Assessments in Evaluating Primary Health Care Quality: Multicenter, Cross-sectional Pilot Study in 7 Provinces of China

**DOI:** 10.2196/40082

**Published:** 2022-12-02

**Authors:** Minrui Zeng, Yiyuan Cai, Jin Cao, Qianyu He, Xiaohui Wang, Yun Lu, Huijuan Liang, Dong Xu, Jing Liao

**Affiliations:** 1 Department of Medical Statistics & Epidemiology School of Public Health Sun Yat-sen University Guangzhou China; 2 Department of Epidemiology and Medical Statistics School of Public Health Guizhou Medical University Guiyang China; 3 School of Public Health Zhejiang University School of Medicine Hangzhou China; 4 Department of Preventive Medicine & Maternal and Child Health School of Public Health Guizhou Medical University Guiyang China; 5 Research Institute for Health Policy of Inner Mongolia Inner Mongolia Medical University Hohhot China; 6 Center for World Health Organization Studies Department of Health Management School of Health Management of Southern Medical University Guangzhou China; 7 ACACIA Lab for Implementation Research Southern Medical University Institute for Global Health Dermatology Hospital of Southern Medical University Guangzhou China

**Keywords:** virtual patient, unannounced standardized patient, primary health care, primary care, quality assessment, quality improvement, scenario, simulation, simulate, medical education, cross-sectional, digital health, eHealth

## Abstract

**Background:**

The unannounced standardized patient (USP) is the gold standard for primary health care (PHC) quality assessment but has many restrictions associated with high human and resource costs. Virtual patient (VP) is a valid, low-cost software option for simulating clinical scenarios and is widely used in medical education. It is unclear whether VP can be used to assess the quality of PHC.

**Objective:**

This study aimed to examine the agreement between VP and USP assessments of PHC quality and to identify factors influencing the VP-USP agreement.

**Methods:**

Eleven matched VP and USP case designs were developed based on clinical guidelines and were implemented in a convenience sample of urban PHC facilities in the capital cities of the 7 study provinces. A total of 720 USP visits were conducted, during which on-duty PHC providers who met the inclusion criteria were randomly selected by the USPs. The same providers underwent a VP assessment using the same case condition at least a week later. The VP-USP agreement was measured by the concordance correlation coefficient (CCC) for continuity scores and the weighted *κ* for diagnoses. Multiple linear regression was used to identify factors influencing the VP-USP agreement.

**Results:**

Only 146 VP scores were matched with the corresponding USP scores. The CCC for medical history was 0.37 (95% CI 0.24-0.49); for physical examination, 0.27 (95% CI 0.12-0.42); for laboratory and imaging tests, –0.03 (95% CI –0.20 to 0.14); and for treatment, 0.22 (95% CI 0.07-0.37). The weighted *κ* for diagnosis was 0.32 (95% CI 0.13-0.52). The multiple linear regression model indicated that the VP tests were significantly influenced by the different case conditions and the city where the test took place.

**Conclusions:**

There was low agreement between VPs and USPs in PHC quality assessment. This may reflect the “know-do” gap. VP test results were also influenced by different case conditions, interactive design, and usability. Modifications to VPs and the reasons for the low VP-USP agreement require further study.

## Introduction

Improving primary health care (PHC) services is one approach to increasing universal health coverage [1]. PHC provides comprehensive essential health care to the community by supporting access to health monitoring, diagnosis, and treatment [2] in an efficient and cost-effective manner [3,4]. PHC service quality is an important factor affecting population health outcomes and should be strengthened as part of health care system reforms [5,6] and in the face of the drastic challenges of the COVID-19 pandemic.

The unannounced standardized patient (USP) is regarded as the gold standard to assess the quality of PHC services [7-10]. USP is a rigorously trained actors portraying patients with certain diseases who anonymously visit PHC services; they can provide a standardized and timely evaluation of health care providers’ performance that prevents the Hawthorne effect, that is, changes in practice associated with being observed [11]. However, USP is limited to clinical conditions that have no obvious signs and that do not require invasive examinations [12]; they are also difficult to deploy in low- and middle-income countries due to their heavy reliance on personnel and resources. The virtual patient (VP), an improvement on computerized clinical vignettes [13], has been proposed as a potential low-cost alternative to USP. VP is a software tool; they simulate real clinical scenarios and have been widely used in medical education [14] due to their low requirements for equipment, high interactivity, safety, and capacity for repeatable actions [15].

It is unknown whether assessments of quality based on VP agree with those based on USP. Prior studies mainly applied VP in medical education [16-18] as a tool to train students’ clinical thinking, skills in medical history collection and diagnosis [19], and attitudes toward patients [20]. Only a few studies have directly compared VP and standardized patients; these studies have found that skills training was less effective with VPs than with standardized patients as the educational tool [21]. No study so far has used VP for PHC quality assessment. Although VP can examine users’ medical knowledge (ie, their competency) in a similar way as vignettes, the results may not accurately reflect the actual performance of users in real clinical practice [22-24], and the Hawthorne effect cannot be avoided. There is some evidence that VP user interfaces and usability may influence VP-based assessment outcomes [14,25]. The extent to which a VP may serve as a quality assessment tool needs further research [26,27].

The current study belongs to a family of studies of PHC quality assessments in China based on the multicenter, nation-wide ACACIA (Health Care Quality Cohort in China) study [28]. This was a pilot study that specifically aimed to (1) examine the agreement of VPs and USPs in assessing the quality of PHC services and (2) identify factors influencing VP-USP agreement.

## Methods

### Study Design and Procedure

This multicenter, cross-sectional pilot study is part of the ACACIA family of studies. The ACACIA protocol has been published previously [28,29]. Briefly, ACACIA aims to develop and validate USPs and paired VPs to assess clinical quality, cost, and patient experiences in PHC across China. The study sample’s representativeness was ensured by its multistage, clustered sample design [30], stratified by the average life expectancy in each province, geographic variations, and feasibility [31]. Altogether, 7 provinces were selected, and their capital cities and prefecture-level municipalities were used as a stratum; 5 townships or urban subdistricts were selected in each city based on probability proportional to size sampling. PHC facilities were then examined in each location. For this study of the agreement of VPs and USPs, a convenience sample was selected of urban PHC facilities in the capital cities of the 7 study provinces, with USP visits to these centers conducted between 2019 and 2021. All PHC providers in these centers, including licensed practicing clinicians and unlicensed clinicians under supervision of licensed physicians, served as our study population.

Altogether, 720 USP visits were conducted. On-duty PHC providers who met our criteria were randomly selected for USP visits. The PHC providers who were visited by the USPs received a VP assessment of the same cases at least a week later to prevent the practice effect [32]. The agreement between these 2 tests was analyzed with the concordance correlation coefficient (CCC) and the weighted *κ*.

### Ethical Approval

Ethical approval was obtained from the Ethics Committee of Sun Yat-sen University (2017-007), and all PHC providers participating in the VP tests provided informed consent.

### USP and VP Case Selection and Design

The USPs and VPs shared identical case designs to ensure consistency and simplify the development process. The selection and development process for these case designs was reported previously [33]. Case designs were selected based on whether the disease in question (1) had a high frequency of PHC clinical encounters, (2) had a significant disease burden, (3) was present in the main areas of PHC in China, and (4) was feasible for use in a USP test (ie, it was without obvious physiological signs and had a low risk of needing invasive tests). Twelve case designs were selected and rigorously developed: angina, asthma, diarrhea, cold, gastritis, hypertension, lower back pain, migraine, postpartum depression, stress urinary incontinence, tuberculosis, and type 2 diabetes. The validity of the case designs was verified, and they were found to have scale-level content validity indices over 0.90, role-playing fidelity over 90%, and checklist completion accuracy of 88% [34]. Most case designs had 5 modules: medical history, a physical examination, laboratory and imaging tests, diagnosis, and treatment. There were exceptions in 4 case designs (hypertension, lower back pain, migraine, and postpartum depression) that did not require laboratory or imaging tests. Due to the COVID-19 pandemic, tuberculosis cases were excluded to protect the USPs from unnecessary physical examinations, potential harm, and conflict [34]. Thus, only 11 case designs were used in this pilot study. Details of the case design development, modification, and validity testing are provided in Multimedia Appendix 1.

### USP Training and Implementation

The USP actors all received at least one week of competency-based online-offline training and were assessed by specialists who were not members of the research team [34]. Before the site visits, the USP actors were further examined to ensure they could accurately portray the case designs according to the standardized training manual [30]. On the day of the visit, each USP was companied by a facilitator, who pretended to be a relative of the USP. The visits were audio recorded with a hidden recording device; these recordings were also used to monitor the performance of the USP actors and ensure checklist quality. If audio recordings were not available, field reports on what the providers said and did during the visits were upload to the online database of REDCap (Vanderbilt University) immediately after the visit to reduce recall bias. An example of a REDCap entry is provided in Multimedia Appendix 2.

### VP Platform and Implementation

The VP was hosted on an online platform that could be accessed via a mobile phone or computer. The 5 VP modules used 3 different interface designs. For the medical history and diagnosis modules, the PHC providers were required to search for keywords with at least 2 characters to trigger relevant inquiries for selection. The physical examination module displayed all possible options. In the laboratory and imaging test module and the treatment module, some general options (eg, ordering blood tests or electrocardiograms) could be chosen directly, while specific options were made available after searching for keywords. All actions were recorded and uploaded online automatically.

For the field testing, PHC providers who agreed to participate in the VP tests received the VP install package for their mobile phone or personal computer alongside a user demonstration video. For each PHC provider, the cases for the VP test were the same as those for the USP test. The VPs were masked to avoid bias due to providers noticing the tested cases. The VP tests included a training VP case, which allowed the PHC providers to become familiarized with the operation of the system. There were no time limits for any of the VP tests to avoid underestimated results caused by a lack of proficiency. To facilitate the use of the VPs, some tests were organized on-site, which may have led to test results that differed from those completed by the PHC providers independently. Thus, the location and manner of the tests, as well as the number of VP tests assigned to each PHC provider and the age and sex of the providers, were recorded for analysis.

### Outcome Measures

The F1 score, recall, and precision were used to measure the continuity of physical examinations, laboratory and imaging tests, and treatment [35]. However, precision and F1 score could not be calculated for medical history due to missing records for unnecessary consultations during USP visits. We used a method adapted from previous studies [36,37] in which recall represents the proportion of PHC providers who completed the checklist based on clinical guidelines, while precision was used to quantitatively assess unnecessary actions in clinical practice. The F1 score considered both recall and precision to be of equal importance and combined them. As shown in the following equation, the F1 score reflected both recall and precision:




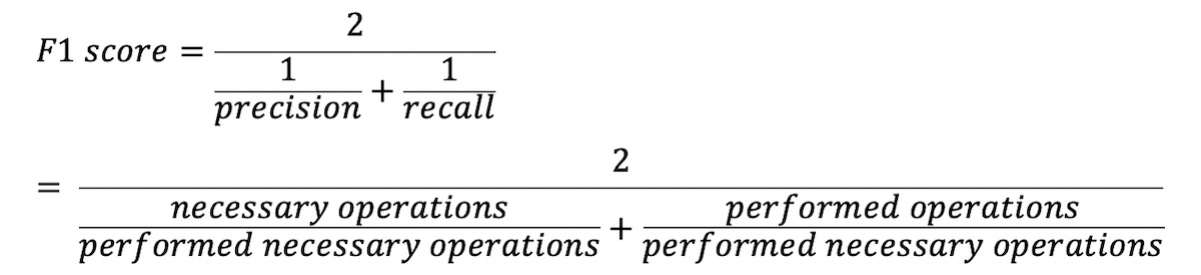




In the equation and in Table 1, recall represents the proportion of necessary actions that were performed in the tests and precision represents the proportion of performed actions that were necessary.

The results of the diagnoses were classified as ordinal variables in line with clinical guidelines and were rated as completely correct, partly correct, or incorrect.

**Table 1:** Explanation of the relationship between test results and case design for virtual patients and unannounced standardized patients. Recall is the number of performed necessary actions divided by the number of necessary actions, while precision is the number of performed necessary actions divided by the number of performed actions.

	Performed actions	Unperformed actions
Necessary actions	Number of performed necessary actions	Number of missing necessary actions
Unnecessary actions	Number of performed unnecessary actions	N/A^a^

^a^N/A: not applicable.

### Statistical Analysis

Characteristics of the PHC providers and VP test information are shown as the mean (SD) for continuous variables and percentages for categorical variables. CCC, which reflects the criterion validity of the VP tests [28], was used to analyze the agreement between precision, recall, and F1 score for VP and USP tests. CCC values <0.90, 0.90 to 0.95, 0.95 to 0.99, and >0.99 were considered to represent poor, moderate, substantial, and almost perfect agreement, respectively [38]. The weighted *κ* (square weighted) was used to analyze diagnostic agreement [28]; weighted *κ* values <0.20, 0.20 to 0.40, 0.40 to 0.60, 0.60 to 0.80, and >0.80 were considered to represent poor, moderate, substantial, good, and almost perfect agreement, respectively [39,40].

Multiple linear regression was used to identify factors influencing VP-USP agreement. Using the VP tests as the dependent variable and USP tests as the independent variable, several multiple linear regression models were established, and the models were stepwise adjusted according to cases, characteristics of the PHC providers (ie, age, sex, and city), and test conditions (ie, test deployment and number of tests). Significant covariates in these models were controlled jointly in a fully adjusted model. Partial regression coefficients of the USP tests are reported. Statistical analysis was carried out using the R (version 4.0.5; R Foundation for Statistical Computing) packages stats (version 4.0.5), Desc Tools (version 0.99.43), and psych (version 2.1.9).

## Results

### Characteristics of PHC Providers and VP Test Information

**Figure 1:**
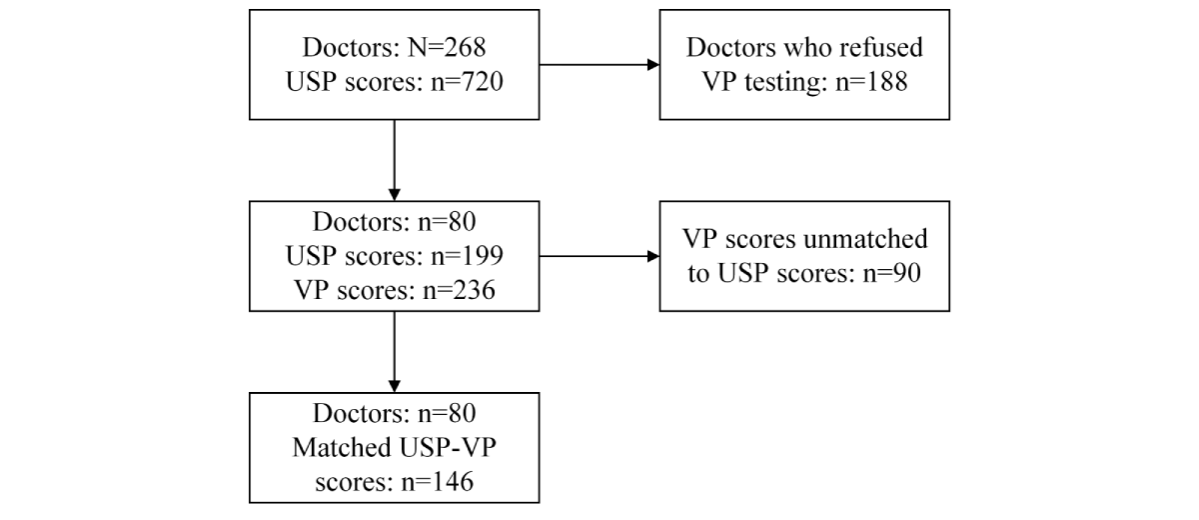
Flow chart of the recruitment process. USP: unannounced standardized patient; VP: virtual patient.

The recruitment process is shown in Figure 1. Of 268 PHC providers who were visited by USPs, 80 agreed to conduct the VP sessions, yielding 236 valid VP scores. However, only 146 VP scores could be matched with the original USP scores.

The characteristics of the PHC providers included in the analysis were as follows: over 80% (67/80) were between 30 and 50 years old and most were male (48/80, 60%). About 40% (35/80) of the PHC providers worked in Guangzhou. Test deployment type for the VP tests was mainly field-testing (59/80, 74%) and more than half (42/80, 53%) of the PHC providers were tested by a single case. The average VP test time was 13.49 (SD 9.33) minutes. The most frequently tested case design was low back pain, with 25 tests. The least frequently tested cases were asthma, gastritis, migraine, and postpartum depression, with 7 tests each. Details are shown in Tables 2 and 3.

**Table 2:** Characteristics of primary health care providers (N=80).

Categories		Values, n (%)
**Age** **(years)**		
	<30	5 (6)
	30-50	67 (83)
	≥50	8 (10)
**Sex**		
	Male	48 (60)
	Female	32 (40)
**Location (city)**		
	Changsha	12 (15)
	Xi’an	7 (9)
	Guangzhou	35 (44)
	Lanzhou	8 (10)
	Hohhot	11 (14)
	Guiyang	5 (6)
	Chengdu	2 (3)
**Case designs**		
	Angina	9 (6)
	Asthma	7 (5)
	Gastritis	7 (5)
	Cold	16 (11)
	Type 2 diabetes	18 (12)
	Diarrhea	13 (9)
	Hypertension	13 (9)
	Low back pain	25 (17)
	Migraine	7 (5)
	Postpartum depression	7 (5)
	Stress urinary incontinence	24 (16)

**Table 3:** Virtual patient test situations (N=80).

Categories		Values, n (%)
**Test deployment**		
	Field-testing	59 (74)
	Self-testing	21 (26)
**Number of tests**		
	1	42 (53)
	2	22 (28)
	≥3	16 (20)

### Agreement Between VP and USP Tests

Test outcomes and CCCs for the medical history, physical examination, laboratory and imaging tests, and treatment modules are listed in Table 4. The USP test results showed high precision (over 0.47), but the VP test results showed varying degrees of degradation (ranging from 0.25 to 0.51), which resulted in very poor agreement. Recall for medical history and treatment was similar for the USP and VP tests. It is worth noting that the physical examination module and the laboratory and imaging test module had results for the USPs that were nearly 3 times higher than for the VPs. All the CCCs for recall were poor. The F1 score and its CCC were close to the recall values, except for the CCC for physical examination. The weighted *κ* for diagnosis was 0.32 (95% CI 0.13-0.51), which was unsatisfactory. Details for the weighted *κ* are shown in Multimedia Appendix 3.

**Table 4:** Test outcomes and concordance correlation coefficients. Precision and F1 score could not be calculated for unannounced standardized patients for medical history due to missing consultation records.

Test modules	Precision (SD)				Recall (SD)				F1 score (SD)		
	USPs^a^	VPs^b^	CCC^c^ (95% CI)	USPs		VPs	CCC (95% CI)	USPs		VPs	CCC (95% CI)
Medical history^a^	—^d^	0.51 (0.35)	—	0.19 (0.15)		0.13 (0.13)	0.37 (0.24 to 0.49)	—		0.18 (0.16)	—
Physical examination	0.47 (0.50)	0.25 (0.30)	0.13 (0.01 to 0.26)	0.11 (0.15)		0.34 (0.31)	0.04 (–0.05 to 0.13)	0.17 (0.21)		0.20 (0.19)	0.27 (0.12 to 0.42)
Laboratory and imaging tests	0.47 (0.49)	0.45 (0.34)	0.21 (0.03 to 0.38)	0.18 (0.20)		0.57 (0.36)	–0.06 (–0.15 to 0.03)	0.25 (0.27)		0.43 (0.27)	–0.03 (–0.20 to 0.14)
Treatment	0.77 (0.41)	0.45 (0.48)	0.07 (–0.06 to 0.20)	0.21 (0.18)		0.20 (0.25)	0.24 (0.10 to 0.39)	0.31 (0.23)		0.26 (0.31)	0.22 (0.07 to 0.37)

^a^USP: unannounced standardized patient.

^b^VP: virtual patient.

^c^CCC: concordance correlation coefficient.

^d^Not available.

### Factors Influencing VP-USP Agreement

To explore factors that affected VP-USP agreement, we used multiple linear regression. For medical history, there was a significant correlation between VP and USP scores that remained stable after adjustment (ranging from 0.32 to 0.34, *P*<.001). In contrast, despite factor adjustment, the correlation between the VP and USP scores was not significant (*P*=.74) for the laboratory and imaging test module. The correlation was significantly weakened after adjusting the cases for the physical examination and treatment modules. Details are listed in Multimedia Appendix 4.

Using stepwise variable selection in the fully adjusted model, all the correlations between VP and USP scores became weaker after adjustment. The partial correlation coefficients were 0.314 (95% CI 0.183-0.445) for recall for the USPs for medical history; 0.071 (95% CI –0.090 to 0.023) for F1 score for physical examination; –0.025 (95% CI –0.169 to 0.118) for F1 score for laboratory and imaging tests; and 0.045 (95% CI –0.133 to 0.223) for F1 score for treatment. Furthermore, for medical history, female sex (versus male) and Changsha and Lanzhou (versus Guangzhou) were negatively associated with recall for VPs, while test time was positively associated with recall for VPs. The F1 scores for the physical examination module and the laboratory and imaging test module were only associated with case design. The F1 score for treatment was only associated with cases and the cities where the PHC providers worked. Combining the results of these models revealed that the major influencing factors were case design and city. Details are shown in Table 5.

**Table 5:** The association between assessments using virtual patients and unannounced standardized patients using stepwise regression for each module.

Test modules				*β* (95% CI)		*P* value		Standardized *β*
**Medical history**								
	Recall for USPs^a^		.314 (.183 to .445)		<.001		.351	
	Female sex		–.049 (–.089 to –.009)		.02		–.366	
	**City**							
		Guangzhou	0 (ref)					
		Changsha	–.067 (–.126 to –.009)		.03		–.506	
		Lanzhou	–.065 (–.129 to –.001)		.049		–.489	
	Test time		.002 (.001 to .004)		.006		.205	
**Physical examination**								
	F1 score for USPs		.071 (–.090 to .023)		.39		.080	
	**Case design**							
		Low back pain	0 (ref)					
		Cold	.203 (.100 to .306)		<.001		1.086	
		Gastritis	.169 (.028 to .311)		.02		.907	
**Laboratory and imaging**								
	F1 score for USPs		–.025 (–.169 to .118)		.74		–.025	
	**Case** **design**							
		Low back pain	0 (ref)					
		Cold	.206 (.074 to .337)		.003		.768	
		Stress urinary incontinence	–.269 (–.401 to –.137)		<.001		–1.005	
		Type 2 diabetes	–.289 (–.416 to –.161)		<.001		–1.079	
		Gastritis	–.386 (–.543 to –.228)		.003		–1.440	
**Treatment**								
	F1 score for USPs		.045 (–.133 to .223)		.62		.034	
	**Case** **design**							
		Low back pain	0 (ref)					
		Cold	.432 (.300 to .563)		<.001		1.404	
		Hypertension	.419 (.283 to .555)		<.001		1.363	
		Type 2 diabetes	.350 (.223 to .477)		<.001		1.138	
		Postpartum depression	.173 (.003 to .344)		.05		.564	
		Gastritis	–.176 (–.347 to –.006)		.05		–.573	
		Stress urinary incontinence	–.189 (–.301 to –.078)		.001		–.615	
		Migraine	–.198 (–.374 to –.023)		.03		–.645	
	**City**							
		Guangzhou	0 (ref)					
		Lanzhou	–.190 (–.299 to –.082)		<.001		–.619	
		Xi’an	–.178 (–.313 to –.042)		.01		–.577	

^a^USP: unannounced standardized patient.

## Discussion

### Principal Findings

Our study examined the agreement between using VPs and USPs to assess the quality of PHC in China. We found that the agreement between VP and USP results was low in general, which may result from the “know-do” gap. The VP tests might also have been influenced by different case conditions, different interface designs of the VPs, and the usability of the VPs.

We found that the agreement between VP and USP scores was low in our study sample. The USP scores were low in terms of recall, indicating that our study participants performed only some of the necessary actions, especially for the physical examination module and the laboratory and imaging test module. This suggests that PHC providers only partially performed the guideline-recommended checklist items in actual practice, which might be the result of a lack of incentives or limited time and resources [5,41,42]. In contrast, the VP scores showed relatively high recall scores, with module-specific variation, compared to the USP scores. One possible explanation for the low agreement is the “know-do” gap [43-45]. The USP tests assessed how the PHC providers performed in real clinical practice with evidence-based indicators [43,46]. However, the VP testing was more likely to assess whether the providers knew how to use their knowledge, which is defined as their competency [43]. With unlimited time and resources, the PHC providers might have performed extra actions to exclude other diseases, even more than required for differential diagnosis. Thus, VP testing may be more akin to examinations in a medical training setting and therefore indicate the competence of the examinees, whereas USP testing may be more likely to assess the quality of care in actual practice [17,47]. Similar findings have been reported by previous studies of medical education, which found that examinees in VP tests tended to explore all possible information [14]. Therefore, despite our intention to use the interactive VPs as an alternative to USPs as a quality assessment tool [17], our current findings on the agreement between VP and USP test results are insufficient to provide strong evidence for such a substitution.

Further analysis using multiple linear regression suggested that VP-based performance varied with case design, indicating that PHC providers’ competency differed with clinical case design. Specifically, variations for type 2 diabetes and gastritis were observed for the test modules and low scores were observed for laboratory and imaging tests for both case designs, while higher scores were seen for treatment for type 2 diabetes than for gastritis. This finding may indicate that PHC providers were more familiar with type 2 diabetes, which is commonly seen in PHC and has a distinctive medical history and physical signs. As a result, laboratory and imaging tests for type 2 diabetes were more likely to be omitted, while appropriate treatment was more likely to be conducted in the VP tests. In contrast, PHC providers might prefer to conduct simple physical examinations for the symptoms of abdominal pain, but they were reluctant to conduct the complex laboratory and imaging testing and treatment that should be offered in accordance with the clinical guidelines for gastritis [48].

Furthermore, the VP interface and usability also influenced the VP testing. By and large, 2 types of interface were used in the VP testing: searching for keywords for consultation for medical history and selecting from multiple choices for physical examinations (for other modules, a mixture of both interface formats was adopted). Specifically, our results showed that the recall score for medical history for the VP testing was two-thirds of the score for USP testing, while the corresponding VP score for physical examination was more than twice that of the USP score. These findings indicate that multiple choices might provide more hints, allowing PHC providers to guess a correct action more easily [14,26]. Although searching for keywords for consultation or actions leads to less bias than the hints provided by multiple choices, this interface might decrease the usability of VPs, particularly when the interaction does not provide enough options or fuzzy searches. Many of the PHC providers in the study found this interaction was not user-friendly and that the consultation questions needed were often not retrievable. Frustrated by the poor interface, they tended to suddenly end the consultation and even drop out of the VP testing entirely. Although searching for keywords decreased usability, this interface should be used for the purpose of quality assessment, albeit with modifications. The influence of the study city may reflect differences in the attitude and capacity for digital adaptation of the PHC providers; those from developed regions with wider use of digital information systems may be more receptive to digitalized medical practice than their counterparts from less-developed regions [41,49]. VP usability may also be influenced by the digital adaptation attitude of PHC providers. Previous studies have found that examinees who are more open to digital innovation, better educated, and younger are more enthusiastic about using and completing digital device–based programs [50,51] like VP testing. Due to missing information on sociodemographic characteristics, our study could not examine the statistical significance of variations in these characteristics other than the city where the participants were located. However, we did find that the agreement between VP and USP results was lower in Changsha, Lanzhou, and Xi’an.

### Study Limitations

The study had several limitations. First, as a purposive sampling approach was used, our research sample may be more likely to have included PHC providers who were receptive to technological innovations, and the extent to which our findings apply to providers who are less receptive needs verification. Although our user experience analysis showed promising results, only a few participants answered the user experience questionnaire. Second, due to substantial missing data for the sociodemographic characteristics of the PHC providers, we failed to identify any remaining influence of these factors on the agreement between VP and USP scores. Third, although we found that the VP interface was a key factor influencing the VP testing, we did not perform a direct comparison of different interfaces with the same disease module. Last, we used a summary score for each module to indicate the providers’ performance, assuming that individual consultation or action items had equal importance. Nevertheless, a hierarchic order may exist among consultation and action items that is specific to the disease conditions under consideration, such that a weighted score may have been better suited for quantifying the providers’ performance [52].

### Study Implications for Further Studies

Our findings highlight the need for further modifications to the VP platform. To improve the design of the VPs to bring them as close as possible to real clinical conditions, strict testing time limits should be implemented to enhance the sense of time pressure. Besides this, the interactive design of the VPs should opt for keyword searching over multiple choices to minimize hints. The creation of clinical settings and the application of keyword searching can be enhanced via advanced technologies, such as virtual simulation, voice input, and fuzzy retrieval [12,53], for a better, user-centered experience [54].

Moreover, to facilitate the implementation of VPs, an add-on program to widely used social software such as WeChat would be preferable to a separate application that requires installation. A short demonstration (of less than 5 minutes) of the main action steps of the VP should be embedded in the program and shown as a mandatory preview for first-time users. If needed, initial training with VP cases should also be provided, so that users can become familiar with the platform.

To better understand the agreement between VP and USP testing, future studies would benefit from systematically collecting information on potential factors contributing to differences between VPs and USPs, using both quantitative and qualitative approaches. For instance, the preferences of PHC providers for different VP interfaces could be examined with questionnaires or interviews to assess differences in perceived authenticity, cognitive load, and motivation [19]. Moreover, potential reasons for the adherence of PHC providers to clinical guidelines and associated influencing factors need to be further explored using mixed methods based on the Theoretical Domains Framework, structured questionnaires, or focus groups [55-57].

### Conclusion

The agreement between VP and USP testing for PHC quality assessment was low. This low agreement may mainly reflect the “know-do” gap, while the VP test results were also influenced by different case conditions, interface design, and usability. To improve VP usability in the resource-limited settings found in PHC, VPs should be modified to be more user-centered, paying attention to the balance between enhancing usability and avoiding hints. Factors influencing the agreement between VP and USP testing need further study.

## Appendix

Multimedia Appendix 1 Case development, modification, and validity.

Multimedia Appendix 2 Example of REDCap.

Multimedia Appendix 3 Classification of diagnosis.

Multimedia Appendix 4 Regression result of different adjustment.

## Abbreviations

CCC: concordance correlation coefficient
PHC: primary health care
USP: unannounced standardized patient
VP: virtual patient

## Data Availability

Due to data safety and property rights, the data sets generated and analyzed during the current study are not publicly accessible but are available upon request to the corresponding author.
