# Supplementary material for: The Agreement Between Virtual Patient and Unannounced Standardized Patient Assessments in Evaluating Primary Health Care Quality: Multicenter, Cross-sectional Pilot Study in 7 Provinces of China
Source: J Med Internet Res. 2022 Dec 2;24(12):e40082. doi: 10.2196/40082 (PMC9758641; doi:10.2196/40082)
Supplement: Multimedia Appendix 1 [file jmir_v24i12e40082_app1.docx]

**Case development and modification**

We developed and validated a total of 12 USP cases, including angina, asthma, child diarrhea, common cold, gastritis, hypertension, lower back pain, migraine, postpartum depression, stress urinary incontinence, tuberculosis, and type II diabetes. We selected those conditions based on two national surveys of the common conditions at PHC. The contents of cases were developed based on clinical guidelines and real medical records. The development for each case was carried out by case-specific development teams, which comprised condition expert, epidemiologists, evidence-based researchers, clinical experts from different levels medical institutions, and case coordinators.

Because of the pandemic, the PHC providers were put on high alert for COVID-19-related symptoms. Patients to PHC with a >37.3℃ body temperature would be directed to a specialized fever clinic. Testing for COVID-19 nucleic acid would also be required. To avoid harming USPs and interrupting the COVID-19 response system, we dropped tuberculosis (TB) case as fever was among the symptoms. We retained two other cases with respiratory symptoms (common cold and asthma) after necessary modifications. For the common cold, we updated its quality checklist per the government COVID-19 guideline to include whether the clinician checked USP’s epidemiological history related to COVID-19. For asthma, we strengthened the asthma-related features after one clinician in an asthma USP’s validation visit suspected the asthma case as COVID-19. For all cases, we added a script for the USPs to report no known exposure to the COVID-19 high-risk population over the past two weeks. We implemented all other cases as initially designed.

Table 1: Validity of the USP assessment tool

| Validity | Measures | How | Why matters? | Results |
| --- | --- | --- | --- | --- |
|  |  |  |  |  |
| Content validity of the quality checklist | Scale-level content validity index with averaging  calculation method (S-CVI/Ave) | Agreement of a multidisciplinary expert panel on the relevance of the checklist via a Delphi process | Checklist serves as the evidence-based criteria for the evaluation of the quality | 12 USP cases ranging from 0.92 to 1, >0.90 threshold |
| Fidelity of USP role-playing | Proportion of accurately used lines during an unannounced USP visit | Quality controllers listened to each voice-recording of the USP visit to verify the accuracy of the USP line use^a^ | Consistently and accurately using the lines is critical to maintaining the standardization of the USP visit | Average 94%, >90% criterion |
| Accuracy of checklist completion by USPs | Agreement of checklist items completed between the USP and the experts | Using the checklist completed by experts listening to the voice-recording of the visits as the gold standard^a^ | USPs must accurately recall and identify the details of clinician consultation, exams, diagnosis and treatment | 88% percent agreement |
| Detection of USP | Proportion of USPs detected by the clinicians | Clinicians reported on any suspected USP visits over the past two weeks | Maintaining the fake identity during the visit is critical to avoid the Hawthorn (observation) effect | 0.68%^b^ |

^a^: We required the field team to update the voice recording before 19:00 of the same day of the visit. The quality controller checked the accuracy of the SP rendition of the lines (>90% accuracy required) and the completion of the checklist, and provided the feedback to the field team. The SPs who did not meet the quality requirement would take an online refresher course on scripts before resuming their visit.

^b^: In the development and validation phase, 147 doctors returned survey forms and 25 reported their suspicion of at least one USP visit. However, only one reported suspicion was actually linked with our USP visits.
