# Supplementary material for: The Agreement Between Virtual Patient and Unannounced Standardized Patient Assessments in Evaluating Primary Health Care Quality: Multicenter, Cross-sectional Pilot Study in 7 Provinces of China
Source: J Med Internet Res. 2022 Dec 2;24(12):e40082. doi: 10.2196/40082 (PMC9758641; doi:10.2196/40082)
Supplement: Multimedia Appendix 2 [file jmir_v24i12e40082_app2.docx]

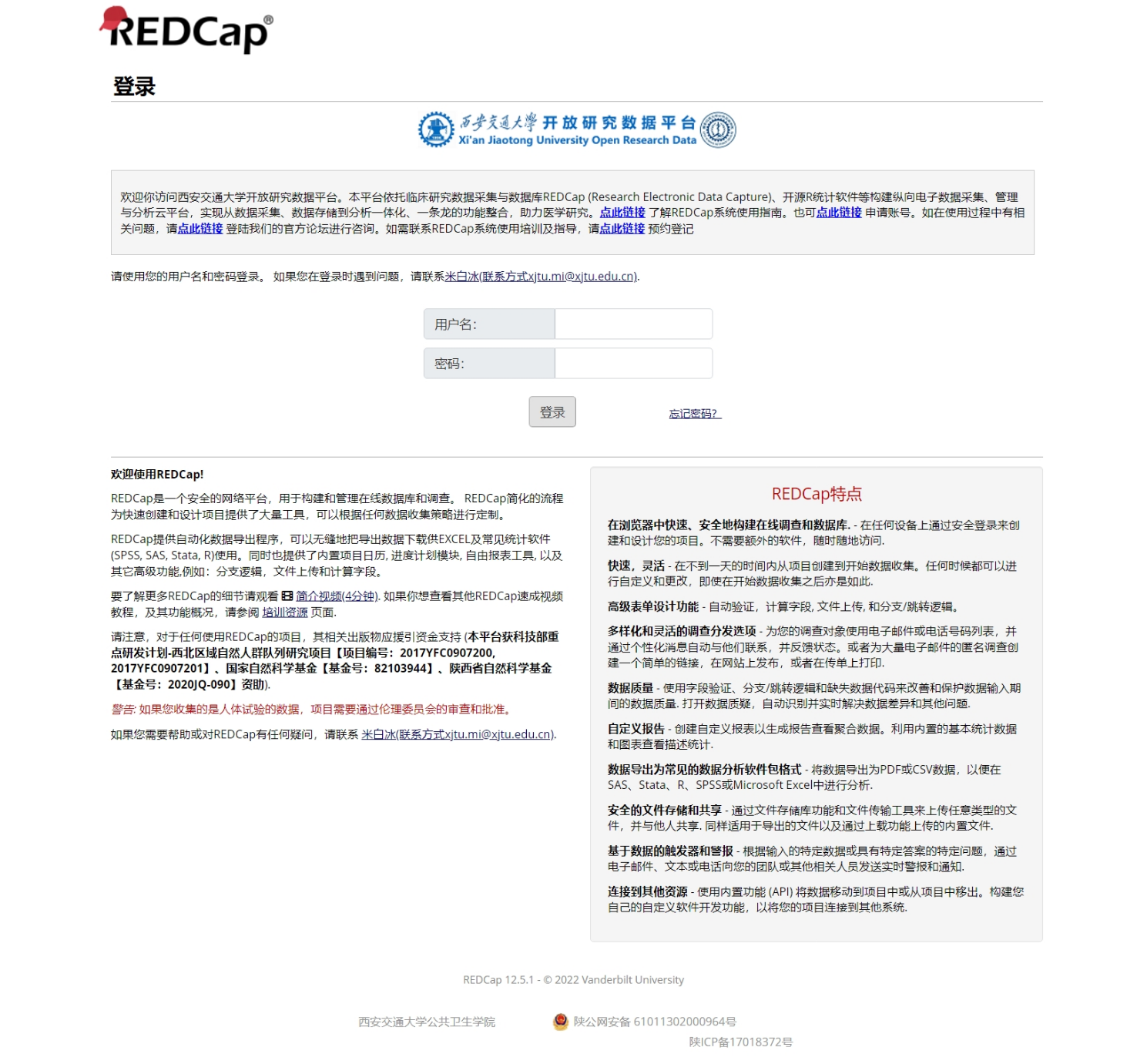


User id

Password

Introduction of REDCap

Figure 1. login page of REDCap


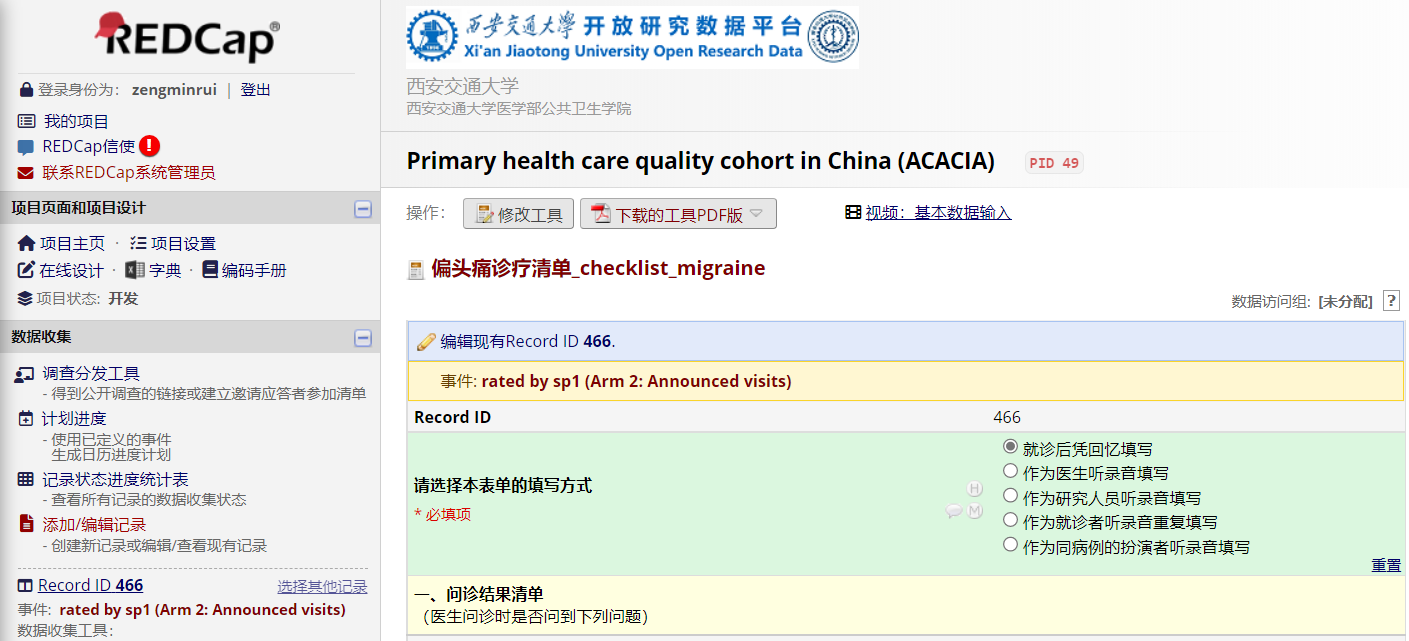


How the checklist was filled?

The checklist for medical history

Figure 2. checklist of migraine
