# Supplementary material for: The Agreement Between Virtual Patient and Unannounced Standardized Patient Assessments in Evaluating Primary Health Care Quality: Multicenter, Cross-sectional Pilot Study in 7 Provinces of China
Source: J Med Internet Res. 2022 Dec 2;24(12):e40082. doi: 10.2196/40082 (PMC9758641; doi:10.2196/40082)
Supplement: Multimedia Appendix 3 [file jmir_v24i12e40082_app3.docx]

**Table 1. Classification of diagnosis**

| **VP** | **USP** | | | |  |
| --- | --- | --- | --- | --- | --- |
|  | **Completely correct** | **Partly correct** | **Incorrect** | **Total** | |
| completely correct | 29 | 9 | 15 | 53 | |
| partly correct | 3 | 2 | 3 | 8 | |
| incorrect | 8 | 5 | 21 | 34 | |
| total | 40 | 16 | 19 | 95**^a^** | |

^a^ Network interruption led to 51 diagnosis results missing.
