# Supplementary material for: The Agreement Between Virtual Patient and Unannounced Standardized Patient Assessments in Evaluating Primary Health Care Quality: Multicenter, Cross-sectional Pilot Study in 7 Provinces of China
Source: J Med Internet Res. 2022 Dec 2;24(12):e40082. doi: 10.2196/40082 (PMC9758641; doi:10.2196/40082)
Supplement: Multimedia Appendix 4 [file jmir_v24i12e40082_app4.docx]

**Regression result of different adjustment.**

**Table 2. The association between VP and USP with different adjustments** ^a^

|  | **Medical history** | | **Physical examination** | | **Laboratory and imaging test** | | **Treatment** | |
| --- | --- | --- | --- | --- | --- | --- | --- | --- |
|  | ***β* (95%CI)** | ***P*** | ***β* (95%CI)** | ***P*** | ***β* (95%CI)** | ***P*** | ***β* (95%CI)** | ***P*** |
| **Model 1^b^** | .36 (.23- .50) | <.001 | .25 (.11- .39) | <.001 | -.04 (-.24- .17) | .73 | .31 (.10- .52) | .005 |
| **Model 2^c^** | .34 (.19-.49) | <.001 | .07 (-.09-.23) | .39 | -.02 (-.17-.12) | .74 | .05 (-.13-.23) | .56 |
| **Model 3^d^** | .32 (.19-.46) | <.001 | .23 (.08-.37) | .002 | -.07 (-.28-.14) | .50 | .32 (.10-.54) | .004 |
| **Model 4^e^** | .32 (.19-.45) | <.001 | .24 (.10-.38) | .001 | -.04 (-.24-.17) | .73 | .32 (.11-.53) | .003 |
| **Model 5 ^f^** | .31 (.18-.45) | <.001 | .07 (-.09-.02) | .39 | -.03 (-.17,.12) | .74 | .05 (-.13,.22) | .62 |

^a^ Medical history using recall, others using F1 score

^b^ Model 1: no adjustment

^c^ Model 2: 11 cases were adjusted.

^d^ Model 3: age, gender, and city were adjusted.

^e^ Model 4: test deployment, number of tests, and test time were adjusted.

^f^ Model 5: all factors were adjusted.
